# The importance of investing in data, models, experiments, team science, and public trust to help policymakers prepare for the next pandemic

**DOI:** 10.1371/journal.pgph.0002601

**Published:** 2023-11-30

**Authors:** Richard Grieve, Youqi Yang, Sam Abbott, Giridhara R. Babu, Malay Bhattacharyya, Natalie Dean, Stephen Evans, Nicholas Jewell, Sinéad M. Langan, Woojoo Lee, Geert Molenberghs, Liam Smeeth, Elizabeth Williamson, Bhramar Mukherjee

**Affiliations:** 1 Centre for Data and Statistical Science for Health (DASH), London School of Hygiene and Tropical Medicine, London, United Kingdom; 2 Department of Health Services Research and Policy, London School of Hygiene and Tropical Medicine, London, United Kingdom; 3 Department of Biostatistics, University of Michigan, Ann Arbor, Michigan, United States of America; 4 Centre for the Mathematical Modelling of Infectious Diseases, London School of Hygiene and Tropical Medicine, London, United Kingdom; 5 Department of Infectious Disease Epidemiology, London School of Hygiene and Tropical Medicine, London, United Kingdom; 6 Indian Institute of Public Health, Public Health Foundation of India, Bengaluru, India; 7 Machine Intelligence Unit, Indian Statistical Institute, Kolkata, India; 8 Department of Biostatistics & Bioinformatics, Rollins School of Public Health, Emory University, Atlanta, Georgia, United States of America; 9 Department of Medical Statistics, London School of Hygiene and Tropical Medicine, London, United Kingdom; 10 Department of Non-communicable Disease Epidemiology, London School of Hygiene and Tropical Medicine, London, United Kingdom; 11 Department of Public Health Sciences, Graduate School of Public Health, Seoul National University, Seoul, Republic of Korea; 12 Interuniversity Institute for Biostatistics and Statistical Bioinformatics (I-BioStat), Universiteit Hasselt & KU Leuven, Hasselt, Belgium; McGill University, CANADA

## Abstract

The COVID-19 pandemic has brought about valuable insights regarding models, data, and experiments. In this narrative review, we summarised the existing literature on these three themes, exploring the challenges of providing forecasts, the requirement for real-time linkage of health-related datasets, and the role of ‘experimentation’ in evaluating interventions. This literature review encourages us to broaden our perspective for the future, acknowledging the significance of investing in models, data, and experimentation, but also to invest in areas that are conceptually more abstract: the value of ‘team science’, the need for public trust in science, and in establishing processes for using science in policy. Policy-makers rely on model forecasts early in a pandemic when there is little data, and it is vital to communicate the assumptions, limitations, and uncertainties (theme 1). Linked routine data can provide critical information, for example, in establishing risk factors for adverse outcomes but are often not available quickly enough to make a real-time impact. The interoperability of data resources internationally is required to facilitate sharing across jurisdictions (theme 2). Randomised controlled trials (RCTs) provided timely evidence on the efficacy and safety of vaccinations and pharmaceuticals but were largely conducted in higher income countries, restricting generalisability to low- and middle-income countries (LMIC). Trials for non-pharmaceutical interventions (NPIs) were almost non-existent which was a missed opportunity (theme 3). Building on these themes from the narrative review, we underscore the importance of three other areas that need investment for effective evidence-driven policy-making. The COVID-19 response relied on strong multidisciplinary research infrastructures, but funders and academic institutions need to do more to incentivise team science (4). To enhance public trust in the use of scientific evidence for policy, researchers and policy-makers must work together to clearly communicate uncertainties in current evidence and any need to change policy as evidence evolves (5). Timely policy decisions require an established two-way process between scientists and policy makers to make the best use of evidence (6). For effective preparedness against future pandemics, it is essential to establish models, data, and experiments as fundamental pillars, complemented by efforts in planning and investment towards team science, public trust, and evidence-based policy-making across international communities. The paper concludes with a ‘call to actions’ for both policy-makers and researchers.

## Introduction

The COVID-19 pandemic raised unprecedented challenges for policymakers, who were required to make quick decisions with limited evidence and limited real-time data to guide them. The pandemic identified critical gaps in global data equity and pandemic preparedness. During the pandemic, data paucity and data opacity hindered effective policy-making in most parts of the world. Even in those settings where some timely and relevant data were generated during the pandemic, there have been few randomised controlled trials (RCTs), and assessing the net effect of public health interventions has been a daunting task. To help the public health community prepare for the next pandemic, the aim of this paper is to develop a broad framework that summarises major learnings from the COVID-19 pandemic, and identifies key areas where concerted action and further research is required.

**Fig 1:**
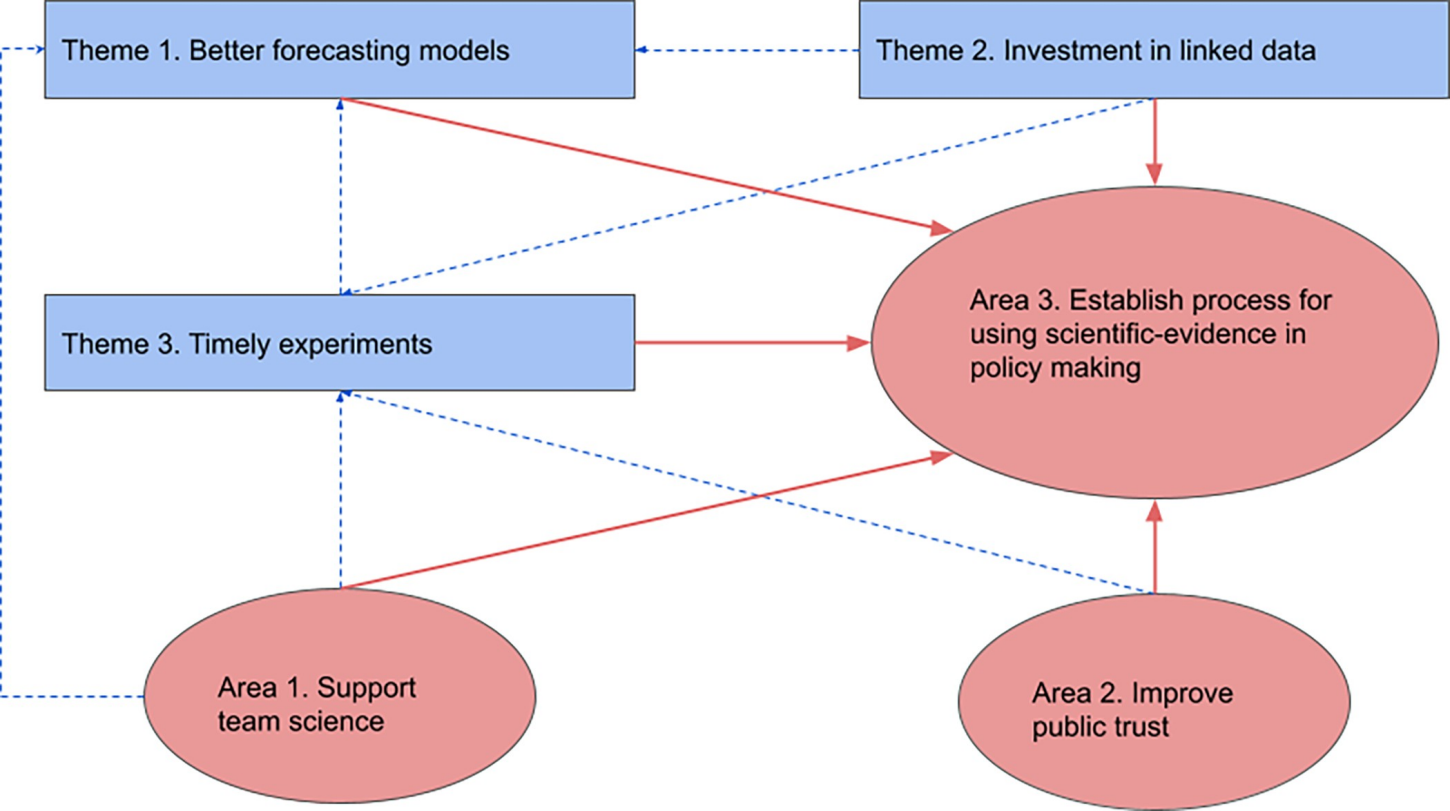
Interlinked themes (1–3) and areas (4–6) that can help preparedness for a future pandemic.

We undertook a narrative review that identified three major themes from the international literature. Each of these themes raised long-term issues for global researchers and policy-makers to address for future pandemic preparedness. These themes are: the challenges of providing accurate forecasts for example of death rates following alternative public policies, the requirement for real-time linkage of health-related datasets, and the role of timely ‘experimentation’ in evaluating interventions. These three themes are distinct from previous reflections [1–6] and were judged of prime importance, particularly for nimble data-driven policy-making towards timely outbreak control. Our narrative review and further reflections also recognised that developing an infrastructure around these themes is insufficient. We also need to invest in three areas that connect to the three main themes and refer to: the need for team science that implies building appropriate teams, that can nimbly pivot to emerging challenges; developing public trust in science, and creating a pathway for translating best use of data, models, and experiments into policy. We outline the conceptual framework in Fig 1.

The paper proceeds as follows. In the next three sections, we review relevant international literature pertaining to each of the three major themes. We then discuss the three interlinked areas, reflect on emerging areas for further research, and finish by offering conclusions and accompanying ‘calls for action’ for researchers and policy-makers.

## Narrative review

### Theme 1: The accuracy and uncertainty of model estimates

#### Challenges in long-term forecasting

At the beginning of an outbreak, decisions must be made under time pressure, usually with little or partial evidence based on incomplete data. In these situations, models that aim to reconstruct recent events, and forecast future ones, can inform urgent decision-making. During the COVID-19 pandemic, public policy was informed by estimates of the current and future rates of infection, hospitalisation, and death, from epidemiological models produced by academic research groups, government agencies, industry teams and individuals. For example, in Belgium [7], model predictions of the number of hospitalisations under different scenarios, informed the timing of restrictive measures, which in turn reduced the number of hospitalisations compared to those predicted in scenarios without ‘policy intervention’. Hence policymakers may make greatest use of such models during the early phases of a pandemic, when despite little information, on, for example, rates of infection, hospitalization, and death, they may provide useful short-term forecasts that inform policy actions. However, faced with a lack of information early in a pandemic, these models may have to make assumptions about policy and behavioural responses, and thus provide potentially inaccurate predictions especially in the longer term. It is therefore vital that the uncertainties, limitations, and assumptions of models are clearly communicated, and used to identify the major gaps in required data, with input parameters updated as soon as possible, ideally by output from accurate surveillance systems. It is also key that the methods used to develop these models are developed further outside of outbreak contexts and robustly evaluated.

#### The benefits of ensemble models

Our literature review uncovered evidence on the relative performance of these models in many countries including Nigeria, Bangladesh, India, South Korea, Poland, Italy, Belgium, Germany, the United Kingdom, and the United States [8–16]. Our review revealed three important insights about the relative performance of alternative modelling approaches. First, these assessments have reported that ensemble forecasts of combinations of models performed better than any one model and were more robust to data quality issues. Cramer et al. [16] compared the predictive accuracy of 90 models forecasting COVID-19-related deaths in the United States. The study found high variation in predictive accuracy across models, locations, and over time. Bracher et al. [13] also found that combinations of models provided more reliable forecasts of the number of cases and deaths in Poland and Germany than individual models. Second, studies also found some models produced relatively accurate forecasts for short time horizons, and in the absence of large-scale government interventions. For example, Jung et al. [12], used ensemble models which provided accurate short-term predictions of the number of COVID-19 cases in South Korea. However, studies reported that models offered less accurate longer-term forecasts, particularly for measures such as the number of deaths [13, 16]. Third, our review identified some of the major challenges in making accurate forecasts over longer time horizons. These challenges related to the novelty of the pathogen itself, but also the complex social, political, and behavioural dynamics within the epidemic.

#### The importance of expert opinion in the context of parameter uncertainty

Even in the absence of policy intervention, it is difficult to predict how behaviour will change as a pandemic progresses. Any retrospective assessment of ‘model accuracy’ must therefore recognise the challenges in anticipating the policy responses and a population’s reaction to them and the pandemic itself. In the presence of policy-changes, models that drew partly on human judgement provided more accurate forecasts, than those that relied solely on epidemiological assumptions [17]. One potential explanation is that expert opinion can more easily capture the potential timing and impact of future policy changes, for example, relaxation of stay-at-home measures. However, the interpretation of results from such models should not imply that models can predict the ‘causal effects’ of alternative policies responses. Rather, use of models is better framed as, ‘predicting outcomes under alternative policy scenarios’. The requisite underlying assumptions behind these models should be clearly spelt out and challenged in extensive sensitivity analyses, with the ensuing uncertainties reported in a full, transparent, and accessible way.

#### Gaps in pandemic modelling

In summary, our literature review revealed that further research is required to explore how these modelling approaches can be generalised to other applications that may differ substantially from the COVID-19 pandemic. The review also identified the need for a more general framework for robust and resilient models and the tools to evaluate their ability to inform decision-makers. Global funding bodies should encourage collaboration rather than competition amongst groups developing models including those with wider skills in statistics, clinical knowledge, behavioural change including anthropology, experimentation, software development, and data science, as this may help in developing ensemble models that provide more accurate and robust forecasts. Such models should be ready for deployment in novel settings. The review also highlighted the need for investment in the data and evidence generation required to quickly populate such models (see next two themes). It is also important for modelling groups to work closely with policy-makers and communicate clearly with the public outside of pandemic periods to help foster trust in models so that they can better inform public policy (see discussion).

### Theme 2: The value of investment in data

The previous section highlighted that during the pandemic a proliferation of models were developed, but to provide accurate, useful forecasts for policy requires real-time data. Countries with reliable population-level data for testing, tracking, sequencing, vaccination, and health outcomes can provide vital insights about for example, the emergence of new variants, the extent to which the effectiveness of a vaccine might wane, or indicative estimates of the effect of policies such as social distancing on transmission rates. During the first wave of the pandemic, very limited information was available on the number of people infected with COVID-19. As the pandemic progressed, more complete information became available from data on positive cases and accompanying health outcomes, demonstrating the vital importance of systems that rapidly, routinely test patients for an emerging virus, and link information on interventions, in particular vaccines, with outcome data. For example, in Israel [18], high-quality studies were conducted to evaluate vaccine waning, with results made immediately available to inform decision-making in other countries.

A challenge many countries faced was to balance data privacy requirements, through measures such as the European Union’s General Data Protection Regulation (GDPR), with the need for timely fine-grained, individual-level data [19]. Our review found examples in Denmark, Germany and the United Kingdom, of National Agency reports summarising the required data [20–22]. In England, the Secretary of State issued NHS data providers and all healthcare organisations, arms-length bodies and local authorities with notices requiring them to process confidential information for COVID-19 purposes; this regulation relating to the control of patient information, was known as ‘the COPI notice’ [23]. However, this directive was only partially successful with some potentially useful COVID-19 data science projects blocked from accessing data, even when covered by COPI notices (see Goldacre report [24]).

National real-time surveillance studies such as the UK Office of National Statistics COVID-19 surveillance study, provided vital additional information [25]. The advantage of a surveillance survey that randomly samples households, is that the sample is not dependent upon patterns of testing and reporting, which can vary by age, occupation, and disease severity. Hence, by including representative strata from the population including those with asymptomatic infection, this survey design can report accurate statistics about the number of true positive cases and prevalence of infection. This information is not only of great use for real-time decision-making, but also to help understand infectious disease transmission both during the current pandemic and into the future.

In many settings national seroprevalence surveys were undertaken only periodically or not at all. Where surveys were completed, they provided useful information, in particular in highlighting that reliance on routine data can lead to substantial under-ascertainment of the true rate of COVID-19 infection and of attributable adverse health outcomes [26]. A major challenge in both LMIC and in developed countries that do not have a national health system, or nationally integrated digital data warehouse, is the fragmented nature of many of the health systems. Hence the required data may only be available across different providers and information systems, and without an established mechanism for linking data, for example linking infection or vaccination status to outcomes [27, 28].

Another investment that provided high value during the COVID-19 pandemic, once basic testing was available, was genome sequencing. This sequencing was helpful in identifying new variants liable to reduce vaccine effectiveness. Since the first whole-genome sequences were made available by China CDC through GISAID on January 10, 2020, over five million genetic sequences of SARS-CoV-2 from 194 countries and territories were made publicly available through GISAID’s EpiCoV database prior to November 2021 [29]. This high-quality, curated data enabled the rapid development of diagnostic and prophylactic measures against SARS-CoV-2 including the first diagnostic tests and the first vaccines to combat COVID-19 as well as continuous monitoring of emerging variants in near real-time. The COVID-19 Genomics UK (COG-UK) initiative at the Wellcome Sanger Institute has sequenced over 2.5 million SARS-CoV-2 genomes (about 20% of the global total) [30].

The genetic sequencing capacities were rapidly scaled rapidly worldwide, recognising that the world is only as strong as the weakest link [31–33]. We note the exceptional success of South Africa, where infrastructure for HIV was developed to generate critical information about genomic sequencing [34]. In countries where this infrastructure does not exist, processing delays can make these data less useful than if available in a timely manner. An ongoing challenge is to sustain the political and public perception of the value of such investments, as even without large paybacks in the interim, these resources will be essential in response to the emergence of genetic variants of SARS-CoV-2, or indeed to future pandemics of new pathogens, not least in helping adapt vaccines. The interlinking of testing, sequencing, vaccination status, and clinical outcomes are critical to feed into multi-strain models for predicting hospitalisation and mortality as well as variant-specific vaccine effectiveness.

The interoperability of data resources both nationally and internationally is required to allow for sharing across public health authorities and jurisdictions. We saw extreme examples of sharing individual data in countries like South Korea, which amended privacy laws after the 2015 MERS outbreak, to accelerate data sharing in any subsequent infectious disease emergency [35]. While it may be debatable as to what extent invasion of private information is appropriate during an emergency, voluntary crowdsourcing of data can often help in to overcome impoverished data infrastructure. Countries with higher levels of public trust are better placed to establish tracking apps for reporting infections in early days of the pandemic which can aid effective surveillance (see also discussion). During the pandemic corporations like Google set up access to mobility data [36], and Facebook/Meta rolled out worldwide surveys [37]. While we need robust official data systems and engagement of data scientists in developing government data action plans, we also require academia-industry-media partnerships. Media outlets such as the Financial Times, the Economist, and New York Times all created excellent dashboards and prediction models during the pandemic. The Johns Hopkins University dashboard became a ‘go to’ source for modelers. A portal to report state-wide COVID data in India (covid19india.org) was established by volunteers and software developers [38]. Going forwards the lessons for crowdsourcing and collaborating on shared data and analytic platforms should inspire us to build on these successes in developing new data destinations and mechanisms for sharing essential information (see also discussion).

### Theme 3: The importance of timely experiments

The requirement to make rapid decisions raised inevitable challenges for the generation of timely evidence. Randomised Controlled Trials (RCTs) provided essential effectiveness evidence to support the use of vaccinations and pharmaceutical interventions, and to stop the rollout of ineffective treatments [39, 40]. The UK was the first country to implement a COVID-19 vaccination programme and quickly introduced new pharmaceutical treatments, once there was evidence of safety and effectiveness [39, 41]. The RCTs that supported these programmes were designed to meet requisite standards and deliver fast results, so those interventions found to be safe and effective, were granted emergency authorisation and made available quickly. This was a highly challenging time for undertaking RCTs, given the policy imperative, and the intense scrutiny from all stakeholders. An area of particular concern was reporting evidence on each vaccine’s side-effects, and carefully interpreting the findings, in particular about the magnitude of the benefits, versus the risk of adverse events according to different baseline characteristics such as age and sex [42, 43].

In some countries, previous investment in clinical research infrastructure were essential to enable RCTs to quickly recruit the large numbers of participants required to generate timely rigorous evidence to directly inform policy. For example, in the UK, previous investment by the National Institute for Health Research (NIHR) in the Clinical Research Network provided essential infrastructure and coordination for successful RCTs undertaken of interventions for COVID-19. For example, the NIHR-funded RECOVERY trial exemplified how this network could facilitate the swift recruitment of patients, to provide practice-changing evidence about drugs such as dexamethasone [41].

In many countries, perennial underfunding coupled with the fragility and fragmentation of many of the healthcare systems, increased the threat posed by COVID-19. Faced with these challenges, the generation of high-quality, context-relevant scientific evidence was essential, and indeed our review found examples of this, for example from the COVID-19 Research Coordination and Learning Initiative (COVID CIRCLE), that directly informed WHO priorities [44]. However, in LMIC, the lack of research infrastructure, effective knowledge sharing from better-resourced countries, sufficient global funding or local prioritisation mean that few RCTs were undertaken related to COVID-19 [45]. In countries such as Ecuador, funding for research was reduced prior to COVID-19, and, during the pandemic, delays due to ethical and regulatory requirements made timely RCTs impractical [45].

Prior to the availability of vaccinations, many countries introduced non-pharmaceutical interventions (NPIs) such as national stay-at-home policies, school closures, travel restrictions, policies of ‘test-trace-isolate’ and mask-wearing, without formal evaluation with RCT or indeed well-designed observational studies. Hence, due to the lack of careful evaluation of NPIs, we are left with a limited evidence base and therefore great uncertainty about the effectiveness of different NPIs, which may differ according to the specific setting. Abaluck et al. [46] report a rare example of an RCT of an NPI, which used a cluster design to evaluate the effectiveness of community-level mask distribution and promotion on COVID-19 infections in rural Bangladesh.

Randomisation raises ethical and practical concerns and may be infeasible for some NPIs such as national stay-at-home policies. There are ethical and practical considerations when health systems are under tremendous stress particularly in undertaking RCTs that require informed consent to be quickly obtained from individual participants. This may lead to greater reliance on observational real-world data during the time of an emerging crisis [47, 48]. More generally there may well be other interventions such as the timing of school closures, or the duration of the isolation period, that have been subject to experimentation. RCTs could inform changes to vaccination programmes, for example, regarding the provision of vaccine boosters for different population subgroups (e.g., according to different age cut-offs). In designing these RCTs it is important to only include subgroups for whom there is uncertainty about the cost-effectiveness of the intervention strategy. The conduct of the trial would have to minimise negative consequences on the general vaccination programme; for example, it would be important to maintain coverage of booster vaccines in those groups for whom the intervention is highly effective and cost-effective in preventing adverse COVID-19 outcomes.

In the absence of RCTs, non-randomised experiments can provide useful evidence about the effectiveness of different forms of NPIs, but must follow basic principles of study design and interpretation, for example in including an appropriate ‘control group’ [49]. In some settings, causal inference approaches such as the synthetic control method can exploit temporal or regional variation in the uptake of interventions. For example, our narrative review found studies that used the variation in the timing of mandatory wearing of face masks across regions in Germany to estimate the impact of mask wearing on rates of infection [50], and the effect of school openings on the spread of COVID-19 contagion in Italy [51]. A retrospective analysis assessed the impact of the timing and the specific forms of NPI undertaken in different parts of India and provided useful context-specific evidence [52]. However, such studies must acknowledge the inevitable assumptions about unobserved confounding, and that results from NPI evaluations, which rely on changing behaviour, may not transport to other settings.

## Discussion

Our narrative review highlighted the crucial role of modelling approaches and interoperable data systems in addressing uncertainty during the pandemic (theme 1). The review also emphasised the need for further investment in data to facilitate the delivery of accurate and timely information (theme 2), and the importance and underuse of RCTs that could identify effective interventions in response to the pandemic (theme 3). Our review also highlights that to prepare for the next pandemic it is essential to adopt a broad framework, and consider the crucial roles of human behaviour, dissemination and communication. We therefore focus our discussion on three additional issues that were highlighted during the response to the pandemic that warrant further consideration, investment and action: these were team science (area 1), public trust (area 2), and the formulation of science-driven policies (area 3). We expand on the importance of each area, drawing examples from the recent pandemic experience, and conclude with calls to action that cross all of the themes and areas.

### Area 1: Team science: Why is it important?

The narrative review of the first theme (models) highlighted that the COVID-19 response relied on a strong multidisciplinary research infrastructure in health data science, which highlighted that the incentives that underline the traditional academic and funding models are not fit for purpose [53]. The novel circumstances of the pandemic catalysed some rapid, effective multidisciplinary collaborations, which epitomised aspects of team science but have not been adopted more widely. A clear example of what team science can deliver is OpenSAFELY, which is a novel platform developed in the UK in response to the pandemic which brought together primary care data of initially more than 30 million people with outcome and COVID-19 test data to undertake rapid population-based COVID-19 research. OpenSAFELY relied on the combined efforts of software engineers, statisticians, mathematical modellers, social scientists, clinicians, and epidemiologists in setting-up and delivering an innovative research platform. The initial work involved the rapid analyses of 17 million records from primary care, to estimate factors associated with COVID-19 deaths within 42 days of initiating the collaboration [54]. This collaboration applied the principles of open science with the real-time creation of reusable and open tools, such as software code.

As part of the pandemic response interdisciplinary teams often came together quite quickly. The WHO assembled technical advisory committees, bringing in experts worldwide to quantify excess deaths due to COVID [55]. Societies like the Royal Statistical Society created a COVID-19 task force, that grappled with nuanced statistical issues, for example regarding methods to reduce bias in the estimation of disease prevalence [56]. In India scientists, clinicians and citizens created an exemplary model by self-organising to share best practices for prevention and treatment [57, 58]. Industry-academic partnerships led to Facebook and Meta rolling out a global survey to understand trends during the pandemic in collaboration with several academic institutions [37]. Rather than rebuilding such collective platforms, we now need to expand interdisciplinary training and support for team science to broader areas where the generation of evidence relies on close coworking and trust amongst multiple stakeholders. Initiatives such as those from The Centres for Disease Control and Prevention (CDC) who funded the first-of-its-kind national network, the Outbreak Analytics and Disease Modelling Network (OADMN) in September 2022 can help, but need to be replicated worldwide to realise the wider benefits from further investments in science.

In future for the principles of open science to be widely applied will require more concrete support from research funders, academic institutions, journals, and panels assessing research quality. The pandemic highlighted the future need for better structures that stimulate and maintain collaborations and promote team science. A pressing issue is that those integral to the process, including early career researchers funded by short-term contracts, must receive appropriate credit. The National Academy of Medical Sciences in the UK has recognised that improving the underlying research infrastructure requires funding panels, peer-reviewed journals and employers, to fully recognise the essential contributions made by each member of the wider research team [59]. Employers’ promotion criteria should recognise a full range of research output including the development of openly shared and reusable software code, communications with media and the public, pre-prints, and reports to government departments and advisors.

So far there has been little progress with incentivising team science, with few funding bodies updating their funding guidelines and models. There are few funding opportunities for funding the required infrastructure in Information Technology, and people such as software engineers with the essential expertise for setting-up and delivering the required research platforms. Academic institutions, research funders and research assessment bodies need to quickly learn the lessons from the pandemic and update their assessment criteria to recognise all aspects of the Open Science process, including, for example, the impacts that follow the curation of open datasets, the development of software code, and the time that teams give to help with the timely, appropriate use of evidence by decision-makers during a pandemic. Ensuring that the requisite research infrastructure in data science is sustained will require changes to institutional cultures which emphasise the value of all contributing disciplines. The biggest impact from investment in team science could be in LMIC where the sharing of knowledge and skills across disciplines is essential to improve local capacity to undertake research of direct relevance for local policymaking [27, 28]. In African countries, we saw exemplary citizen science, with investors, private sector players, institutions, and individuals contributed towards financing the country’s response to the COVID-19 pandemic [60, 61].

### Area 2: The role of public trust in pandemic control

Public trust and confidence in the government, in science, in healthcare providers and in each other are key to battling a pandemic. People are more likely to follow recommendations if they have trust in policymakers and the healthcare system [62, 63]. To increase public trust, it is important to involve the public as stakeholders, form community advisory boards, and recognise citizen science. Transparency and accountability are core principles for policymakers and data, presented lucidly and visually can help with that. As disinformation was flooding the social media and media channels, the role of credible journalists, opinion leaders, public intellectuals, community, and religious leaders became crucial in fighting misinformation.

The success of COVID-19 mitigation strategies, such as vaccination programs, or national stay-at-home policies, rested on high levels of public understanding about their purpose, and of the individual’s role in achieving societal goals, such as preventing transmission. A major concern was to try and mitigate any negative effects on vaccine uptake of misinformation circulated on social media [64]. Das and Mishra [65] developed a theoretical framework in which the design of government policies and the concomitant actions of individuals are mediated by the degree of social trust. Silva et al. [66] modelled human behaviours and the impact of public health policies on the dynamics of the curve of actively infected individuals during a COVID-19 epidemic outbreak, using real data from Portugal as an example.

Agencies such as the Science Media Centre in the UK played a crucial role in helping scientists and the media raise public awareness about, for example, the risks of COVID-19 infection, and the uncertainty of future outcomes following alternative policy scenarios [67]. Examples of ongoing and future uncertainties include the potential for new variants of COVID-19, and the durability of vaccine effectiveness. These issues exemplify the challenge for researchers and policy-makers to communicate such uncertainties in a way that enhances, rather than erodes, public trust in how scientific evidence is generated and used in policy-making.

Trust cannot be earned through communications and public engagement alone, and the pandemic provided some clearer instances, for example, in India and the US, of scientists working closely with community and religious leaders to help improve trust in the use of health data within marginalised communities. In the Netherlands, there were direct links with key religious leaders, representing, for example, Christian, Jewish and Islamic faiths, to help reduce risks of COVID-19 transmission at religious events [68, 69]. An ongoing initiative in the US which brings together patients, caregivers, clinicians, community leaders and scientists to understand, prevent and treat Long COVID is the RECOVER initiative. A related initiative is to address privacy concerns that hinder the wider use of health reporting apps, which can help with reporting statistics that are challenging to estimate accurately from other sources [67]. In preparing for future pandemics, it would be helpful to emphasise scientific communication within future school-level curriculum in particular for science, and to expand opportunities for intersectional fields such as data journalism.

### Area 3: From real-time data to real-time policy

Making timely policy decisions that incorporate the best available evidence, requires interchange and trust between those politically accountable for these decisions, the ‘politicians’, and those with the requisite expertise to generate, synthesise and interpret the best available evidence, ‘the scientists’. A crucial lesson from previous pandemics is that it is essential to have a mechanism for quickly communicating policy questions to scientific experts, and for scientists to refine and answer those questions. In the UK, members of the Scientific Advisory Group for Emergencies (SAGE) and Scientific Pandemic Advisory Group on Modelling (SPI-M) advised the government during previous pandemics. The pre-existence of mutual trust and understanding of urgent policy requirements helped with the quick, clear interchange of policy questions and advice. A major challenge for the advisory groups was to communicate uncertainties in the available evidence. The presence of a scientific expert working within the central government improved trust and understanding across the groups in the nuances of the policy questions, and the uncertainties and complexities in the evidence. These features ensured that it was a two-way process with the policy questions refined in response to initial scientific advice, with timely answers given to these questions, and the most important gaps and uncertainties identified. This process also encouraged political support for investments that could fill gaps in the information required to inform policy.

## Limitations

While this narrative review has uncovered major topic areas that must be addressed to help future pandemic preparedness, it does have some clear limitations. First, we did not undertake a systematic review. Such an approach would be challenging, but in future could be undertaken to supplement the findings reported in this paper. Second, we have focused on three major themes, and also considered a further three complementary areas of high importance. However, it is impossible to cover all those areas of international relevance for future pandemic preparedness. In particular, COVID-19 highlighted wide disparities in the evidence generated across countries with different pre-existing levels of infrastructure in health research. In LMIC, there tended not to be linked data, and there was a lack of funding, resources or expedited ethical or regulatory processes for undertaking RCTs of vaccines or for the repurposing of drug therapies. LMIC often relied on the transfer of evidence from high-income settings, and yet for behavioural-based interventions, effectiveness and cost-effectiveness are heavily context-dependent.

The importance of an established process for using scientific evidence in policy-making should not be underestimated and was largely non-existent in many countries. While WHO has identified global health priorities, and at the regional-level, the Africa Centres for Disease Control and Prevention have led an all-Africa research agenda, this requires funding to ensure rapid implementation. This is urgently needed in other regions such as Latin America, where deaths per capita from COVID-19 are especially high. Hence, there is an urgent need for a regional research agenda, with greater prioritisation for health research funding to build the appropriate research infrastructure, in particular to facilitate RCTs, and the development of systems to collate routine health data across fragmented health systems [70].

## Conclusion and calls to action

The COVID-19 pandemic raises new opportunities for data science and models to generate the timely evidence needed by policy makers. An improved response to any future pandemic will require advanced investment that adopts a broad framework in recognising that models must be informed by linked public health data, evidence on effectiveness from RCTs and well-designed natural experiments, supported through public trust in science, driven by properly supported team science, and facilitated by a fluid process by which public policy draws on timely scientific evidence (Table 1). In countries such as the UK, previous investments in health research helped with the generation of some timely RCT evidence, but also highlighted the need for further investment in linked data on exposures, outcomes, and well-designed natural experiments. In Denmark, Israel, and New Zealand access to linked data on seroprevalence, vaccination status, and clinical outcomes, provided important evidence on, for example, risks of adverse outcomes following COVID-19 infection, which helped inform policy. The effectiveness of NPI strategies tends to be context-dependent, and COVID-19 provided few examples of rigorous evaluations. Hence, there is a future need for more experimentation in the timing and rollout of NPI strategies. This could be facilitated by better guidance for researchers and policy-makers about how to rollout NPI strategies in a way that can still facilitate evaluation during the course of a pandemic.

**Table 1:** Calls to action for researchers and policy-makers that emerged from the narrative review and accompanying reflections.

	Action
1.	Establish a broad conceptual framework of research triangulation that can integrate evidence from models, well-designed RCTs, natural experiments and other observational data
2.	Invest in integrated data sources for surveillance and patient care that can be linked easily with other external data sources
3.	Invest in the infrastructure required to undertake timely RCTs, and non-randomised studies with appropriate designs together with guidance for researchers and policy-makers on how to facilitate rigorous, timely evaluation of NPIs during a pandemic
4	Support and incentivise team science and collaborative platforms outside a time of crisis to help models provide reliable forecasts with appropriate quantification of uncertainty
5.	Recognise that the public are key stakeholders, and that scientists as well as policy-makers have a crucial role in increasing public trust and that citizens have a vital role in raising questions of scientists and policy-makers.
6	Improve scientific communication with governments and the public
7.	Conduct research to develop and evaluate interventions that address structural reasons for health inequalities

This review identifies several initiatives as important for future pandemic preparedness including further global investment in better-linked real time routine data, timely experiments, team science, improving public trust, and in rapidly using evidence for policy-making. The COVID-19 pandemic also highlighted major structural inequities according to sociodemographic characteristics within countries. For example, in England and the United States, two of the countries with the highest rates of excess COVID-19 deaths, the pandemic exacerbated existing inequities in health outcomes. In both these countries minority ethnic groups had excess risks of adverse COVID-19 outcomes compared with White populations [71, 72]. Hence a major priority for further research to develop interventions that address structural reasons for health inequalities is of high priority, and would benefit from linked health, social and employment data within countries, with resultant insights to be shared across international communities [70] (Table 1).
