# Correction: The importance of investing in data, models, experiments, team science, and public trust to help policymakers prepare for the next pandemic

**DOI:** 10.1371/journal.pgph.0003402

**Published:** 2024-06-14

**Authors:** Richard Grieve, Youqi Yang, Sam Abbott, Giridhara R. Babu, Malay Bhattacharyya, Natalie Dean, Stephen Evans, Nicholas Jewell, Sinéad M. Langan, Woojoo Lee, Geert Molenberghs, Liam Smeeth, Elizabeth Williamson, Bhramar Mukherjee

The affiliation for the fourth author is incorrect. The correct affiliation is not indicated. Giridhara R. Babu is not affiliated with #6 but with: Department of Population Medicine, College of Medicine, QU Health, Qatar University, Doha.
